# SHCBP1 drives tumor progression in triple-negative breast cancer

**DOI:** 10.3389/fonc.2025.1587236

**Published:** 2025-07-29

**Authors:** Huiling Wang, Huijuan Dai, Liheng Zhou, Yanping Lin, Wenjin Yin, Jingsong Lu

**Affiliations:** Department of Breast Surgery, Renji Hospital, School of Medicine, Shanghai Jiao Tong University, Shanghai, China

**Keywords:** SHCBP1, single-cell RNA-seq, triple negative breast cancer, proliferation, migration

## Abstract

**Backgrounds:**

Triple-negative breast cancer (TNBC) represents the most aggressive breast cancer subtype. The limited treatment options underscore the urgent need to explore novel molecular targets to combat TNBC progression. This study investigates the oncogenic functions of SHCBP1 in TNBC.

**Materials and methods:**

Bulk RNA-seq and single-cell sequencing (scRNA-seq) data for TNBC samples were acquired from the Cancer Genome Atlas (TCGA) dataset and GSE161529, respectively. SHCBP1 expression at the mRNA and protein levels was compared between TNBC and normal breast tissues. The prognostic significance of SHCBP1 in TNBC was assessed using Kaplan–Meier analysis. The potential biological functions of SHCBP1 were explored through gene ontology (GO), Kyoto Encyclopedia of Genes and Genomes (KEGG), and gene set enrichment analysis (GSEA). Immunofluorescence was utilized to determine the subcellular localization of SHCBP1 during cell division. Quantitative PCR (qPCR) and western blotting were employed to measure SHCBP1 expression in breast cancer cell lines. Subsequently, the impact of SHCBP1 on TNBC cell proliferation and migration was evaluated *in vitro*. Finally, scRNA-seq analysis was conducted to characterize SHCBP1 expression patterns at the single-cell resolution.

**Results:**

SHCBP1 is markedly upregulated in TNBC tissues, and its overexpression is associated with poorer survival outcomes. Functional enrichment analysis reveals that SHCBP1-related genes are significantly enriched in pathways involved in cell-cycle regulation and DNA damage response. *In vitro* studies demonstrate that SHCBP1 enhances TNBC cell proliferation and migration. The scRNA-seq analysis displays the cell clusters in which SHCBP1 is primarily expressed. Cancer epithelial cells exhibiting higher SHCBP1 expression display stronger interactions with stromal cells in the tumor microenvironment.

**Conclusions:**

This study elucidates the critical role of SHCBP1 in TNBC progression, highlighting its potential as a therapeutic target. These findings provide a foundation for further exploration of SHCBP1 in TNBC treatment strategies.

## Introduction

Triple-negative breast cancer (TNBC) accounts for approximately 15%–20% of all breast cancer cases and is recognized as the most aggressive subtype (1). Compared with hormone receptor-positive or HER2-amplified breast cancers, TNBC is associated with increased metastatic potential, higher recurrence rates, and worse clinical outcomes (2). The lack of targeted therapies has made chemotherapy a cornerstone of systemic treatment for TNBC. Therefore, identifying novel molecular targets to overcome TNBC progression and enhance chemosensitivity is critically important.

SHC1 acts as an intracellular scaffold protein for several essential signaling pathways, including MAPK and PI3K/AKT in breast cancer (3). This gene encodes three isoforms—p46SHC, p52SHC, and p66SHC, with p52SHC being the predominant isoform implicated in mammary tumorigenesis (4). SHCBP1 (Shc SH2-domain-binding protein 1) was initially identified as an interacting partner of the adaptor protein p52SHC (5). Emerging evidence highlights the involvement of SHCBP1 in cancer progression across various malignancies. For example, SHCBP1 enhances the migration and invasion of bladder cancer cells by inhibiting RACGAP1-mediated Rac1 inactivation (6). In gastric cancer, after stimulation by EGF, SHCBP1 is translocated into the nucleus and binds to PLK1 to promote the phosphorylation of MISP. Blocking the binding of SHCBP1 and PLK1 can enhance the sensitivity of gastric cancer cells to trastuzumab (7). Similarly, in lung cancer, SHCBP1 promotes migration and invasion and confers resistance to cisplatin-induced apoptosis through Wnt pathway activation (8). In breast cancer, elevated SHCBP1 expression correlates with advanced clinical stages and poor prognosis (9, 10). Despite these significant findings, the functional significance of SHCBP1 in TNBC remains poorly understood, warranting further investigation.

Our study aimed to utilize bioinformatics analysis and *in vitro* experiments to investigate the oncogenic functions of SHCBP1 in TNBC. We found that SHCBP1 was markedly upregulated in TNBC, and patients with higher SHCBP1 expression experienced a worse prognosis. Functional enrichment analyses revealed the correlation of SHCBP1 with the cell cycle and DNA damage pathways. Subsequently, we demonstrated that SHCBP1 positively regulated TNBC cell proliferation and migration *in vitro*. Finally, scRNA-seq analysis was conducted to explore the molecular characteristics of SHCBP1 at the single-cell resolution. Our data highlights the crucial role of SHCBP1 in clinical outcomes and tumor progression in TNBC.

## Results

### SHCBP1 is upregulated in TNBC and correlates with a poor prognosis

**Figure 1:**
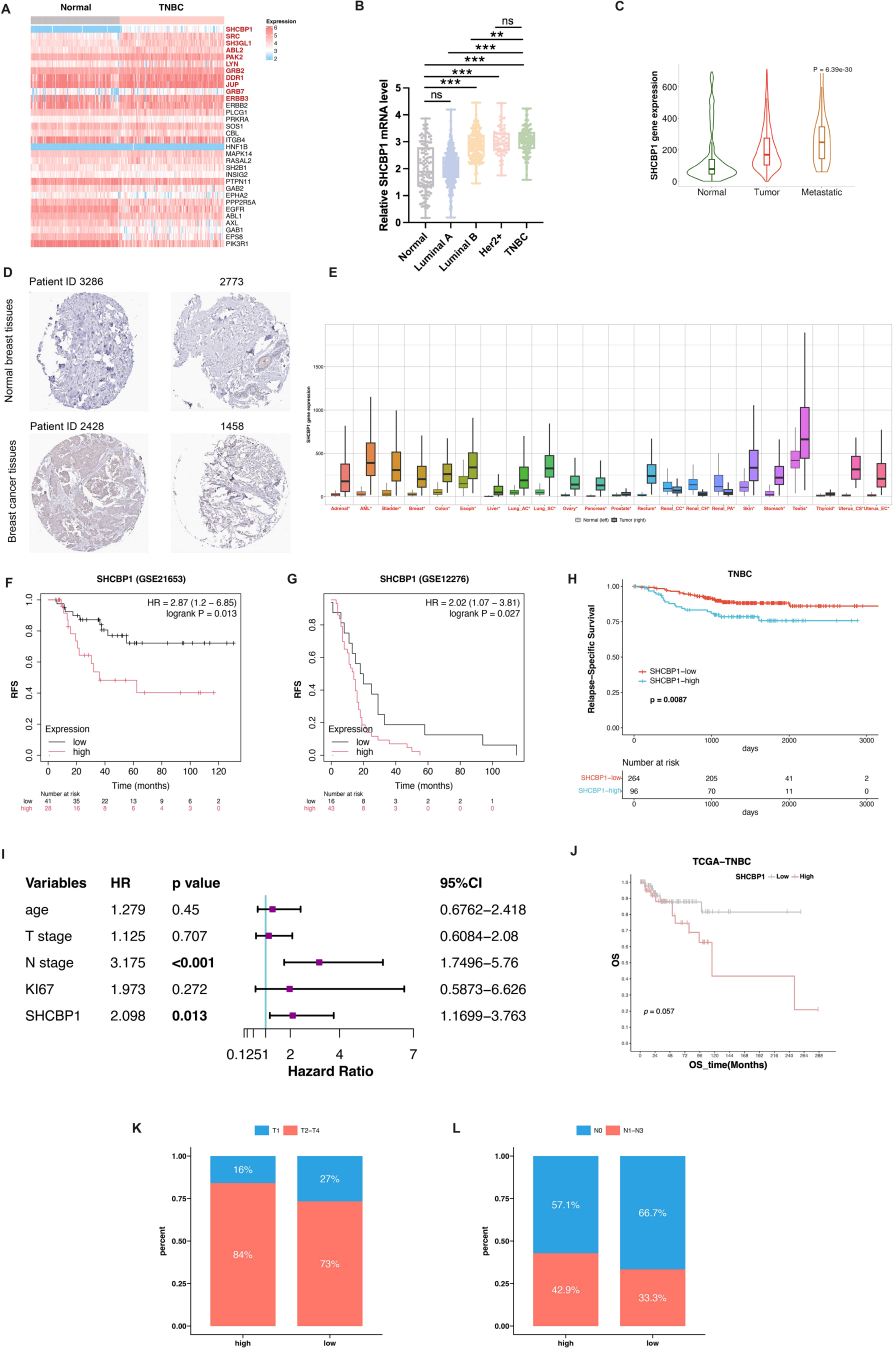
Upregulated expression and prognostic value of SHCBP1 in breast cancer. **(A)** Heatmap analysis illustrating differential mRNA expression profiles of 32 SHC1-binding proteins between TNBC and normal breast tissues from the TCGA dataset. **(B)** *SHCBP1* mRNA expression across different breast cancer subtypes in the TCGA database. **(C)** TNMplot platform analysis demonstrates the expression gradient of *SHCBP1* in normal tissues, primary tumors, and metastatic lesions. **(D)** Immunohistochemical (IHC) images from the Human Protein Atlas (HPA) database show a distinct SHCBP1 protein expression in TNBC and normal tissues. **(E)** Pan-cancer analysis via the online tool TNMplot indicates significant upregulation of *SHCBP1* mRNA in multiple malignancies compared with normal counterparts. **(F, G)** Kaplan–Meier analysis using GSE21653 **(F)**, *p*=0.013) and GSE12275 **(G)**, *p*=0.027) cohorts confirms the association between elevated SHCBP1 levels and decreased relapse-free survival. **(H)** Kaplan–Meier analysis using the FUSCC cohort validates the association between elevated SHCBP1 levels and decreased relapse-free survival (*p*=0.0087). **(I)** Forest plot of multivariate Cox results of the clinicopathological indicators and SHCBP1. **(J)** Kaplan–Meier survival analysis of the TCGA cohort reveals reduced overall survival in patients with high SHCBP1 expression (*p*=0.057). **(K, L)** The distribution characteristics of T stage and N stage in the high- and low-SHCBP1 groups in the TCGA dataset. Data are presented as mean ± SD, *p < 0.05; **p < 0.01; ***p < 0.001; *ns* > 0.05.

According to the findings of Shi et al. (7), SHC1 binds to 32 proteins in gastric cancer cells. We investigated the mRNA expression of the 32 SHC1-binding proteins in TNBC and normal breast tissues by analyzing The Cancer Genome Atlas (TCGA) database. As shown in **Figure 1A**, the mRNA level of *SHCBP1* was predominantly upregulated in TNBC compared with normal breast tissues. *SHCBP1* mRNA expression was significantly higher in TNBC and Her2-positive breast cancer (BC) compared with luminal A and luminal B subtypes (**Figure 1B**). Moreover, *SHCBP1* had a relatively higher expression in metastatic tumors than in primary tumors (**Figure 1C**). To verify our results at the protein level, we used the IHC staining data from the HPA database; the results showed that SHCBP1 protein expression in BC was higher than that in normal breast tissues (**Figure 1D**). By analyzing the mRNA expression of *SHCBP1* in human pan-cancer tissues, we found that *SHCBP1* mRNA expression was also significantly upregulated in many other cancer tissues (**Figure 1E**).

Then, we asked whether SHCBP1 expression impacts survival in TNBC. Kaplan–Meier survival analysis of TNBC patients in GEO datasets showed that patients with a higher SHCBP1 expression tended to have worse clinical outcome (**Figure 1F**, *p*=0.013; **Figure 1G**, *p*=0.027). The result was validated on TNBC patients in the Fudan University Shanghai Cancer Center (FUSCC) cohort (**Figure 1H**, *p*=0.0087). Additionally, multivariate Cox analysis showed that SHCBP1 was an independent prognostic factor (**Figure 1I**, *p*=0.013). A similar trend was also observed in the TCGA dataset (**Figure 1J**, *p*=0.057), indicating that SHCBP1 may exert an oncogenic effect in TNBC. To investigate the correlation of SHCBP1 and clinical parameters, we performed a chi-square test in the TCGA dataset. We found that low-SHCBP1 breast cancer had favorable clinical characteristics, such as more T1 (**Figure 1K**, 27% vs. 16%) and N0 (**Figure 1L**, 66.7% vs. 57.1%).

### Genetic alterations of SHCBP1 in BC and somatic mutation profiles between high-SHCBP1 and low-SHCBP1 expression groups

**Figure 2:**
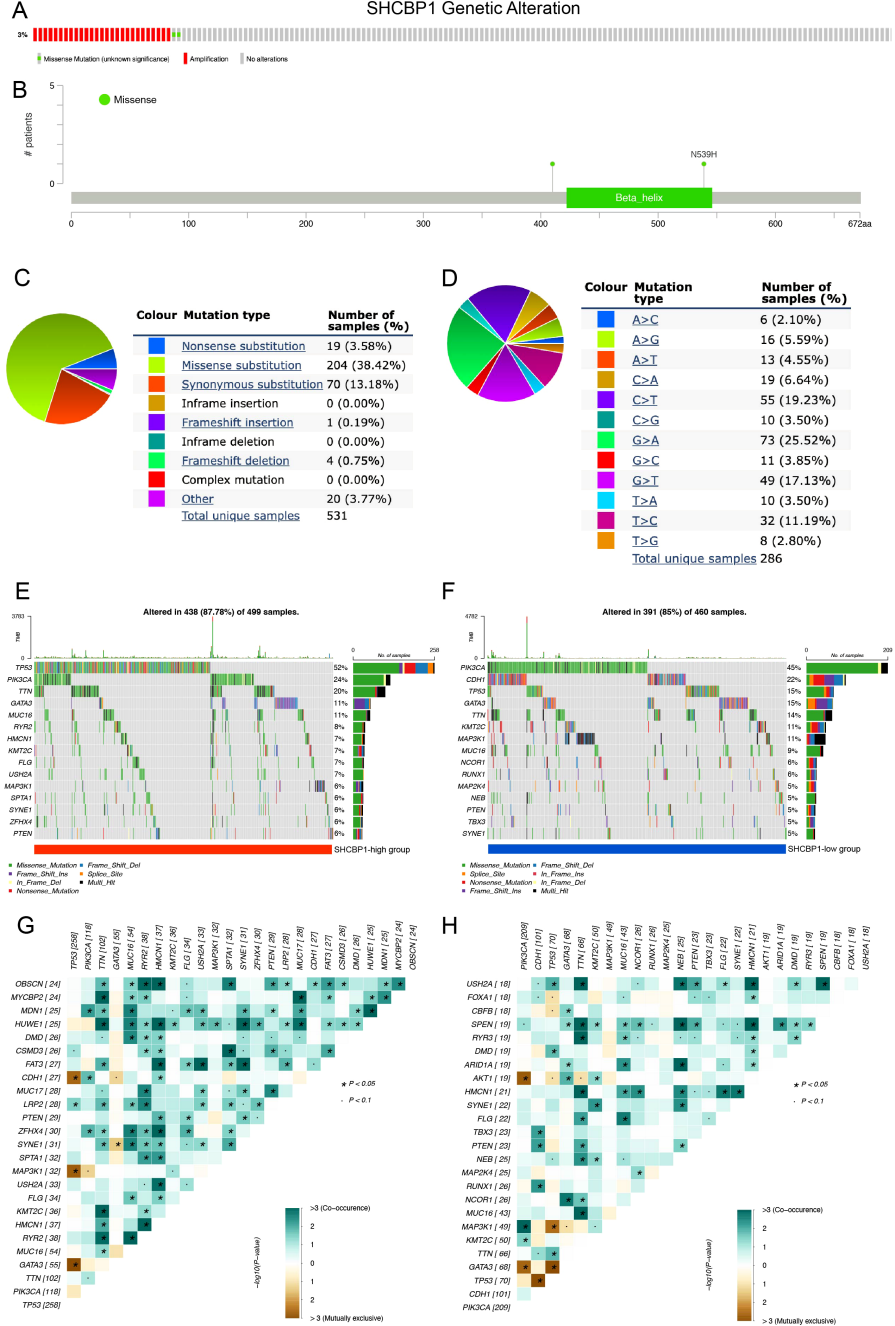
Genetic alterations of SHCBP1 in breast cancer and somatic mutation profiles between high-SHCBP1 and low-SHCBP1 expression groups in TNBC. **(A)** cBioPortal oncoprint illustrating SHCBP1 genetic alterations identified in 963 cases from TCGA-BRCA. **(B)** Domain-specific mutation landscape of SHCBP1 in breast cancer, visualized using cBioPortal. **(C, D)** COSMIC database analysis of SHCBP1 mutation types **(C)** and substitution mutation types **(D)** in breast cancer. **(E, F)** The waterfall plot demonstrates the top 15 most frequently mutated genes in the high-SHCBP1 **(E)** and low-SHCBP1 **(F)** groups in TNBC. **(G, H)** Heatmaps depict the co-occurrence and mutual exclusivity of 25 mutated genes between the high **(G)** and low **(H)** SHCBP1 groups, with green and brown gradients indicating the probability of these events, where darker shades correspond to higher probabilities. *p < 0.05.

We first analyzed the frequency and types of SHCBP1 via the cBioPortal database using the TCGA-BRCA database, containing 963 BC samples with mutation and copy number alteration (CNA) data. In breast cancer, the alteration frequency of SHCBP1 was found to be 3%, with 27 cases showing amplification and 2 cases exhibiting a missense mutation (**Figure 2A**). The mutation landscape details the various types and sites of modifications in the SHCBP1 gene (**Figure 2B**). Following this, we leveraged the COSMIC database to conduct a more thorough analysis of the mutation types. The results revealed that missense substitutions were found in 38.42% of the breast cancer samples, and synonymous substitutions accounted for 13.18% (**Figure 2C**). The predominant base substitutions were G > A (25.52%), C > T (19.23%), and G > T (17.13%) (**Figure 2D**).

To examine the genomic alterations between high- and low-SHCBP1 expression groups, we categorized the somatic mutation data from TCGA-TNBC based on the median expression level of SHCBP1. Interestingly, we found that the somatic mutation landscape varied between the high- and low-SHCBP1 expression groups, indicating distinct biological characteristics between the two groups. In detail, in the high-SHCBP1 group (**Figure 2E**), the top five most mutated genes were TP53 (52%), PIK3CA (24%), TTN (20%), GATA3 (11%), and MUC16 (11%). For the low-SHCBP1 group (**Figure 2F**), the leading mutated genes were PIK3CA (45%), CDH1 (22%), TP53 (15%), GATA3 (15%), and TTN (14%). The heatmap results showed that the occurrence of co-occurring somatic mutations is notably lower in the low-SHCBP1 group compared with the high-SHCBP1 group (**Figures 2G, H**).

### The biological function of SHCBP1 in TNBC is associated with cell cycle and DNA repair

**Figure 3:**
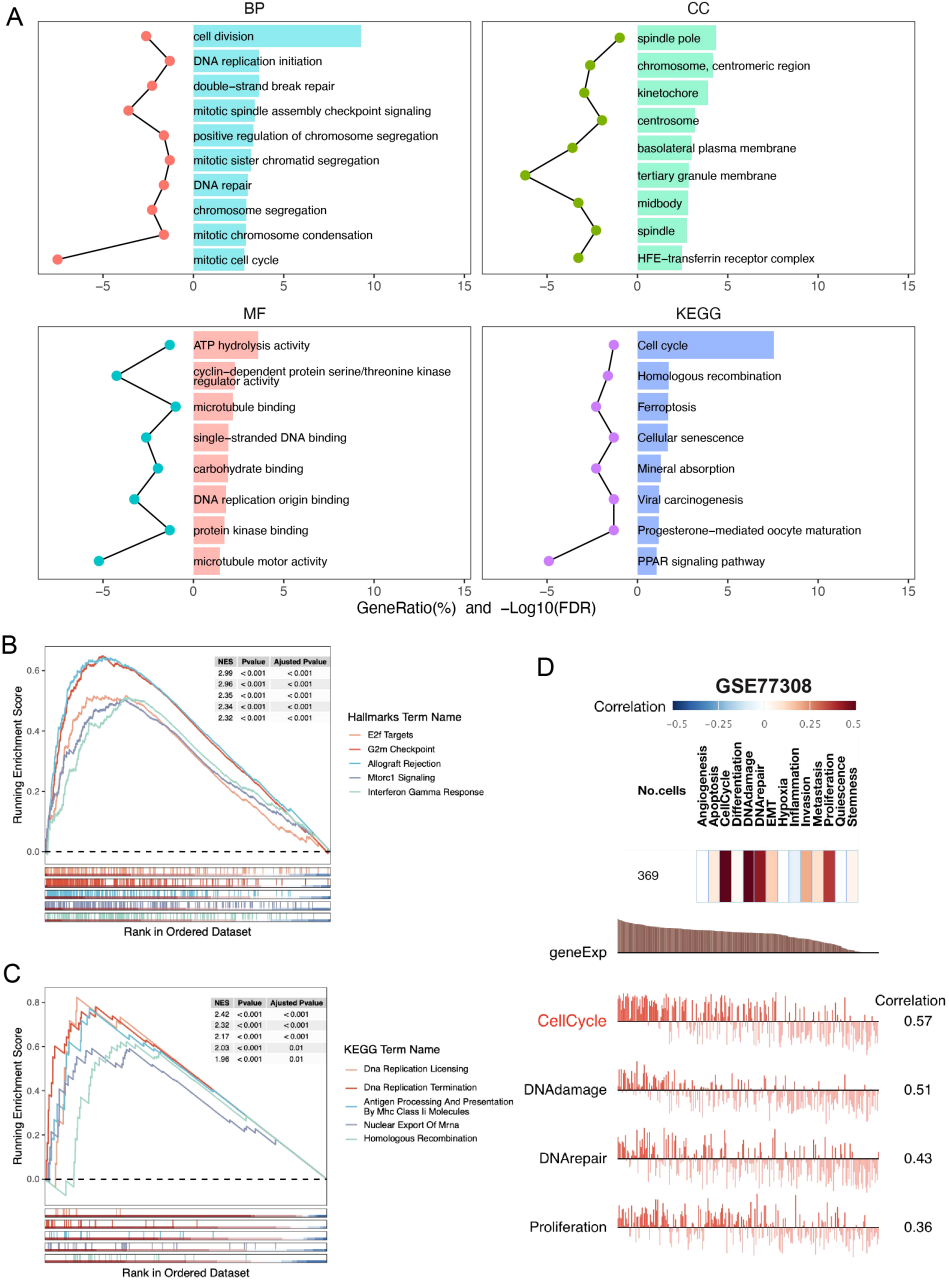
Gene function analysis of SHCBP1 in TNBC based on the TCGA database. **(A)** GO and KEGG enrichment analyses of DEGs (|LogFC| > 1, *p*-value < 0.05) overexpressed in the high-SHCBP1 group. **(B, C)** Visualization of GSEA in HALLMARK **(B)** and KEGG **(C)** from samples with high SHCBP1 expression, respectively. **(D)** The correlation of SHCBP1 with different functional states in BC analyzed by the CancerSEA database.

To determine how SHCBP1 might drive the aggressiveness of TNBC, we initially conducted GO and KEGG analyses. Our findings revealed that the enriched pathways were primarily associated with cell division, DNA repair, spindle and microtubule binding, and homologous recombination (**Figure 3A**). Correspondingly, the GSEA results demonstrated that elevated SHCBP1 mRNA expression was associated with E2F targets, G2/M checkpoints, DNA replication, and homologous recombination (**Figures 3B, C**). Subsequently, to examine the relationship between SHCBP1 and cancer functional states at a single-cell level in TNBC, we analyzed the GSE77308 cohort via the CancerSEA database. **Figure 3D** illustrates that SHCBP1 expression positively correlates with functional states, including the cell cycle, DNA damage, and DNA repair in TNBC. These results further indicate that SHCBP1 might be crucial in the malignant progression of TNBC.

### Subcellular localization and protein expression of SHCBP1 during mitosis

**Figure 4:**
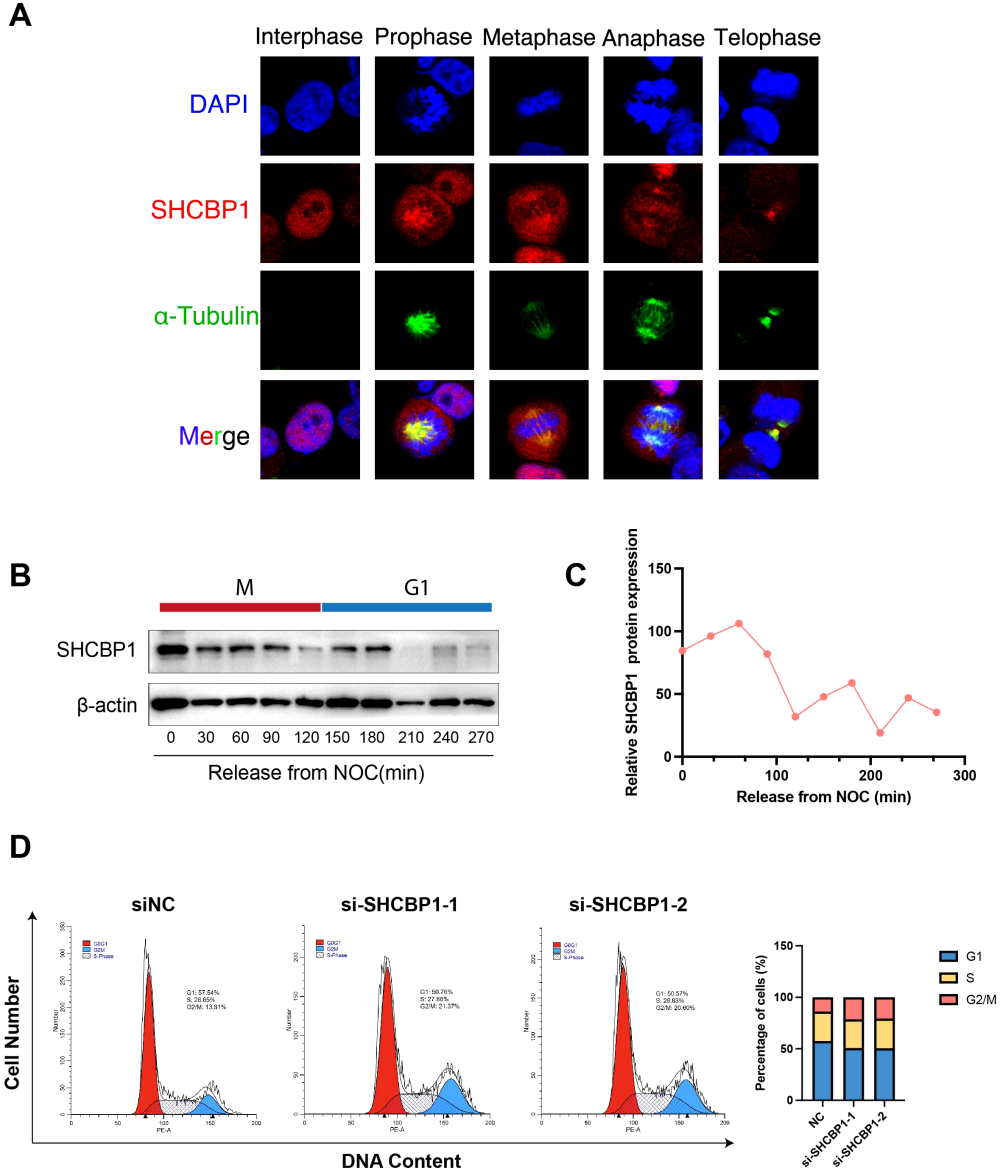
Subcellular localization and protein expression of SHCBP1 during mitosis. **(A)** Subcellular localization of SHCBP1 during mitosis. Immunofluorescence analyses of MDA-MB-231 cells show α-tubulin (green), SHCBP1 (red), and DNA (blue). **(B)** Western blot for SHCBP1 proteins in MDA-MB-231 cells through a time course of 4.5 h following nocodazole release. **(C)** Quantification of SHCBP1 protein expression normalized by β-actin. **(D)** Flow cytometry analysis shows the cell cycle distribution of control and SHCBP1-knockdown MDA-MB-231 cells.

To better understand the relationship between SHCBP1 and the cell cycle, we conducted *in vitro* experiments. Immunofluorescence analysis of MDA-MB-231 cells using an anti-SHCBP1-specific antibody showed that SHCBP1 localized to the nucleus during interphase, to microtubules during prophase, to the spindle and centrosome during metaphase and anaphase, and to the midbody during telophase (**Figure 4A**). To examine SHCBP1 protein expression during mitosis, MDA-MB-231 cells were synchronized with nocodazole. A peak of SHCBP1 protein expression was observed in the M phase (**Figures 4B, C**). After knockdown of SHCBP1 in MDA-MB-231 cells, we observed a significant decrease in G1 phase (*p*<0.001) and a significant increase in the G2/M phase (*p*<0.001) (**Figure 4D**), indicating that SHCBP1 knockdown resulted G2/M cell cycle arrest. These results consistently confirm that SHCBP1 plays a significant role in mitosis, which warrants further investigation.

### Loss of SHCBP1 impairs the proliferation and migration of TNBC cells

**Figure 5:**
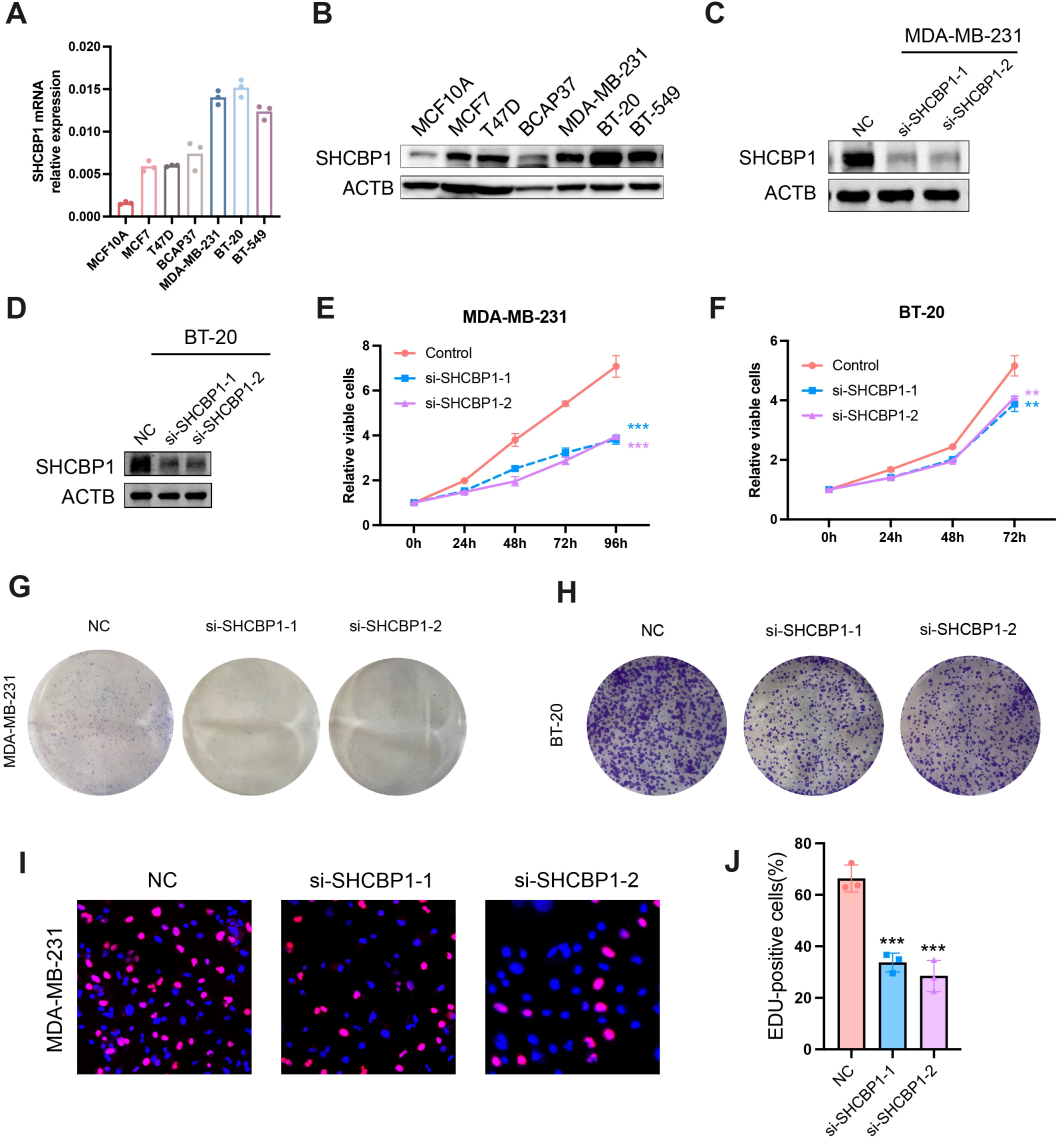
Loss of SHCBP1 impairs the proliferation of TNBC cells. **(A, B)** SHCBP1 mRNA and protein expression in normal breast epithelial cell MCF10A and six breast cancer cell lines, respectively. **(C, D)** The knockdown efficiency of SHCBP1 in MDA-MB-231 and BT-20 cells was confirmed by western blotting. **(E, F)** The proliferation rate of control or si-SHCBP1 cells was assessed by CCK-8 assay. **(G, H)** Representative images of the colony-forming capacity of control or si-SHCBP1 cells. **(I, J)** The proliferation capacity of control or si-SHCBP1 cells was validated by EdU incorporation assay. Proliferating cells were labeled with EdU (red), and cell nuclei were stained with Hoechst (blue). Data are presented as mean ± SD, **p < 0.01; ***p < 0.001.

**Figure 6:**
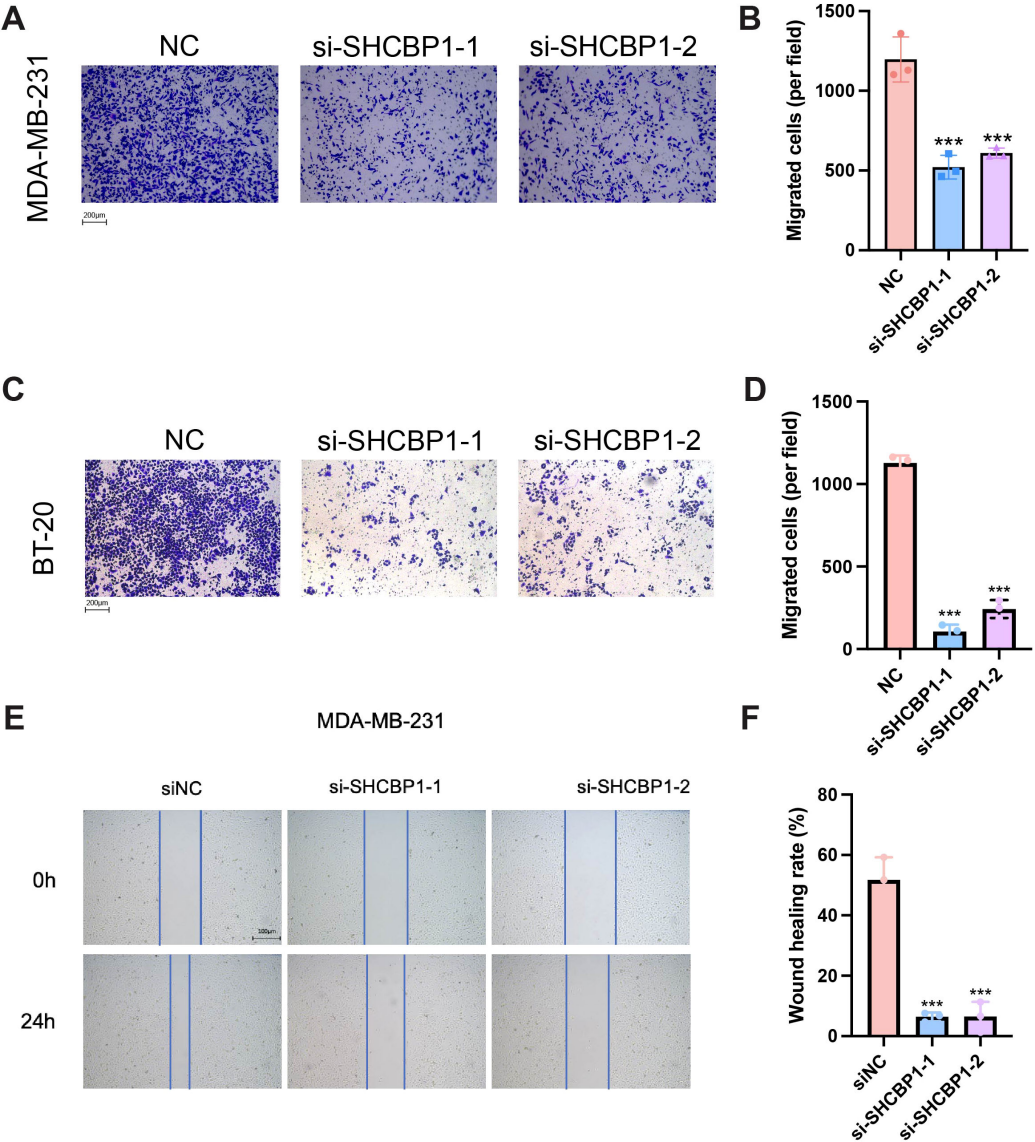
Loss of SHCBP1 impairs the migration of TNBC cells. **(A–D)** The Transwell assay demonstrated that SHCBP1 knockdown significantly inhibits the migration ability of MDA-MB-231 and BT-20 cells. **(E, F)** The wound healing assay results show that the knockdown of SHCBP1 significantly inhibited the migration capacity of MDA-MB-231 cells. Data are presented as mean ± SD, ***p < 0.001.

To explore the role of SHCBP1 in the progression of TNBC, we assessed the impact of SHCBP1 on MDA-MB-231 cell proliferation and migration. Reverse transcription-quantitative polymerase chain reaction (RT-PCR) and western blotting showed that SHCBP1 mRNA and protein levels were elevated in breast cancer cell lines compared with the normal breast epithelial cell line, MCF10A, with TNBC cell lines (MDA-MB-231, BT-20, and BT-549) exhibiting even higher levels (**Figures 5A, B**). Subsequently, we knocked down SHCBP1 in MDA-MB-231 and BT-20 cells using siRNA (**Figures 5C, D**). CCK-8, colony formation, and EdU assays demonstrated that knocking down SHCBP1 significantly diminished the proliferative ability of MDA-MB-231 and BT-20 cells (**Figures 5E–J**). Moreover, the Transwell assay and wound healing assay indicated that SHCBP1 knockdown significantly impaired the migration capability of MDA-MB-231 and BT-20 cells (**Figures 6A–F**). Collectively, these results suggest that SHCBP1 contributes to the proliferation and aggressiveness of TNBC.

### Rescue with SHCBP1 restored the proliferation and migration of TNBC cells

**Figure 7:**
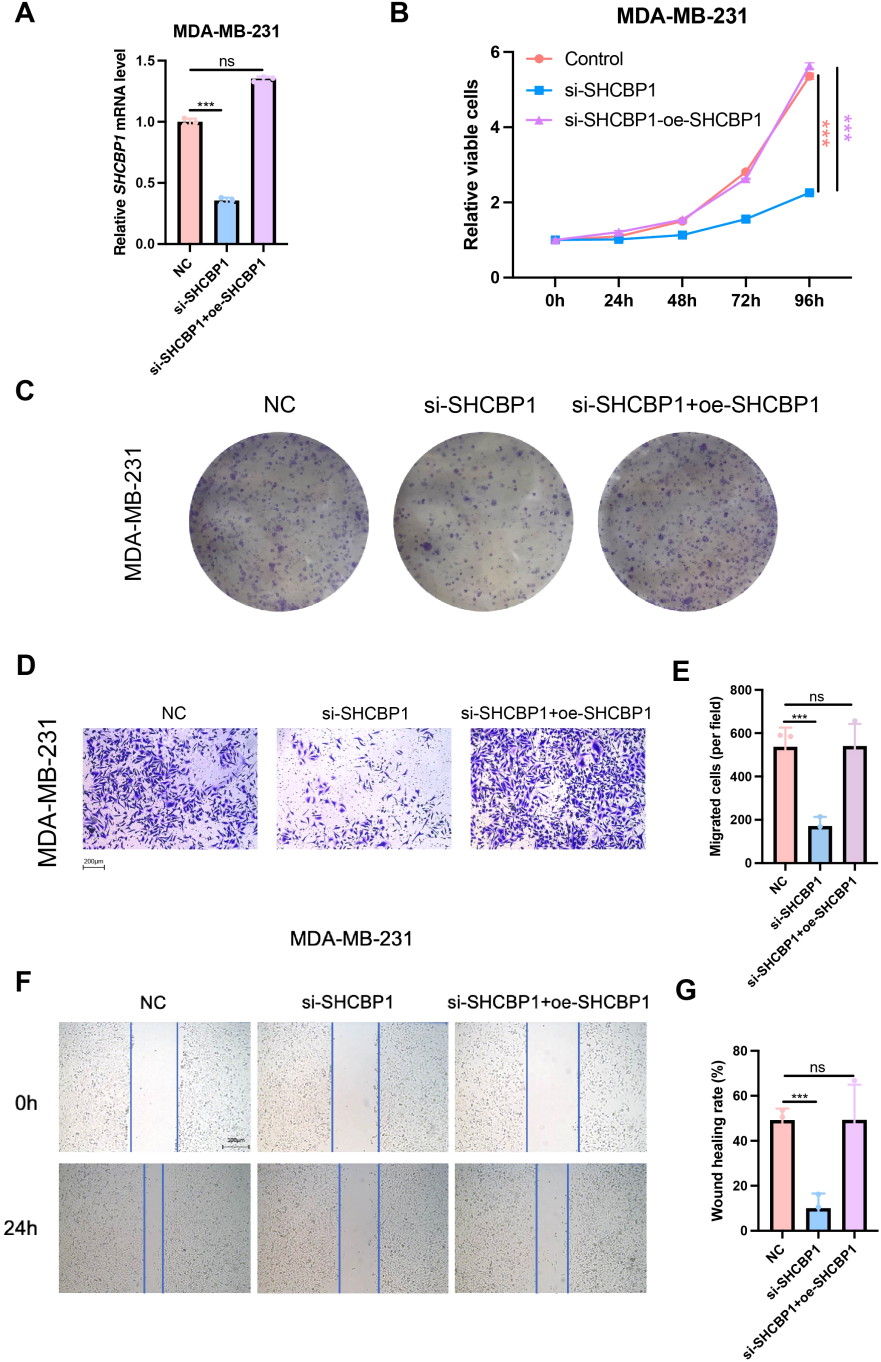
Rescue with SHCBP1 promoted the proliferation and migration of TNBC cells. **(A)** The relative SHCBP1 mRNA level in NC, si-SHCBP1, and the rescue group. **(B)** CCK-8 assays in MDA-MB-231 cells transfected with non-specific control siRNAs (NC), SHCBP1-specific siRNAs (si-SHCBP1), and SHCBP1-specific siRNAs with SHCBP1 overexpression plasmids (si-SHCBP1+oe-SHCBP1). **(C)** Representative images of colony-forming capacity of MDA-MB-231 cells transfected with non-specific control siRNAs (NC), SHCBP1-specific siRNAs (si-SHCBP1), and SHCBP1-specific siRNAs with SHCBP1 overexpression plasmids (si-SHCBP1+oe-SHCBP1). **(D–G)** The Transwell assay and wound healing assay results showed that the overexpression of SHCBP1 rescues the migration capacity of si-SHCBP1 cells. Data are presented as mean ± SD, ***p < 0.001, *ns* > 0.05.

To confirm that the observed proliferation and migration defect was specifically caused by SHCBP1 loss, we re-expressed flag-tagged SHCBP1 in knockdown cells. The relative SHCBP1 mRNA level in NC, si-SHCBP1, and the rescue group was verified by qPCR (**Figure 7A**). CCK-8 and colony formation assays showed that SHCBP1 rescue significantly restored cell proliferation (**Figures 7B, C**). Furthermore, flag-tagged SHCBP1 also rescued the migration impairment in si-SHCBP1 cells (**Figures 7D–G**).

### Molecular features of SHCBP1 at the single-cell level and cellular interaction related to SHCBP1

**Figure 8:**
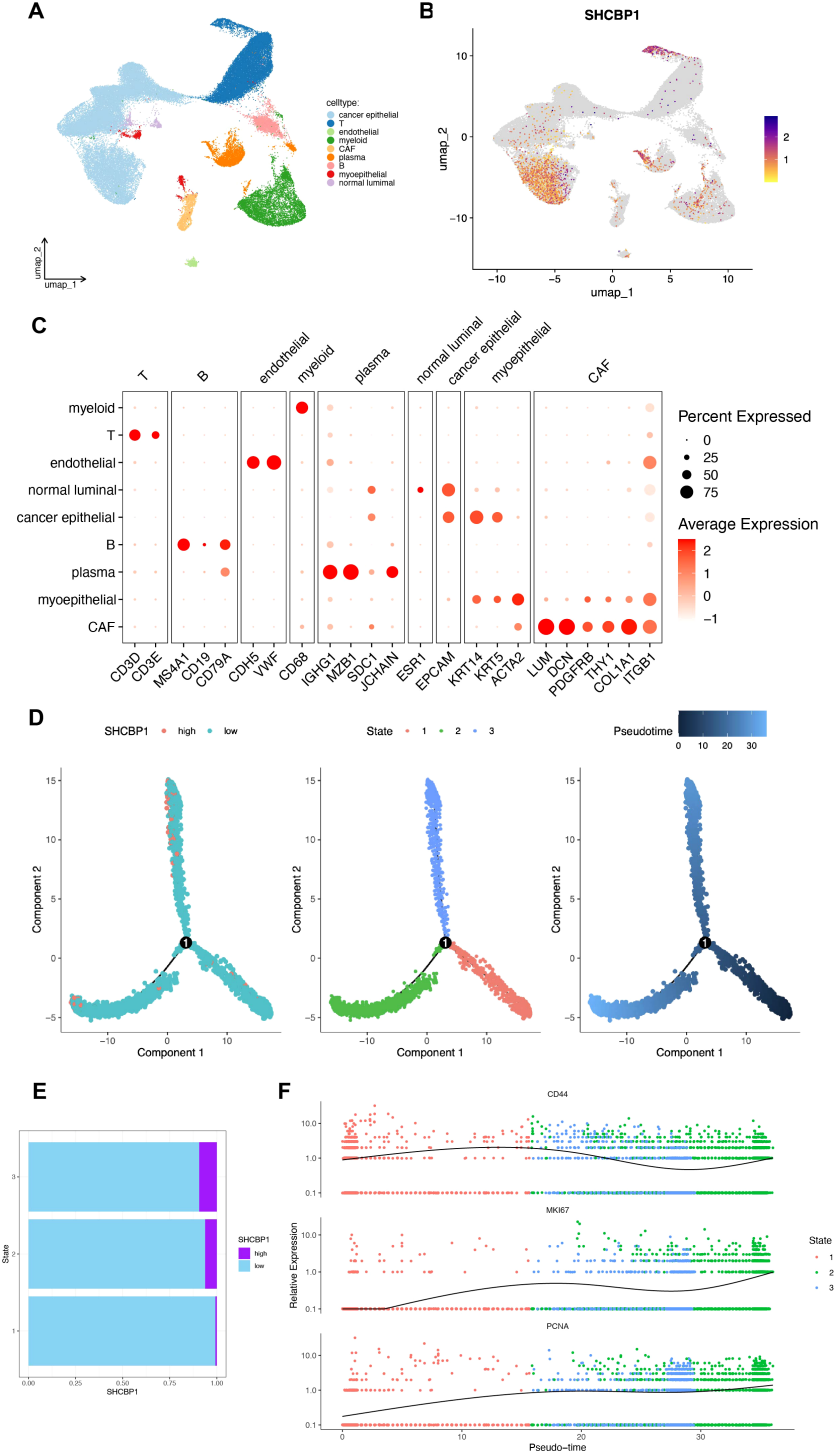
Molecular characteristics of SHCBP1 at the single-cell level. **(A, B)** UMAP for the dimension reduction and visualization of nine cell types **(A)** and visualization of SHCBP1 expression **(B)**. **(C)** Dot plot showing the expression levels of marker genes used to annotate the cell types. **(D)** Pseudotime trajectory analysis based on SHCBP1 expression. **(E)** SHCBP1 expression in three cell states based on pseudotime analysis. **(F)** Pseudotime ordered single-cell gene expression of CD44, MKI67, and PCNA.

Next, we explored the expression characteristics of SHCBP1 in eight TNBC samples using single-cell sequencing analysis. We identified a total of nine cell types (**Figure 8A**). As illustrated in **Figure 8B**, SHCBP1 showed high expression levels in T cells, cancer epithelial cells, myeloid cells, and plasma cells. **Figure 8C** displays the expression of canonical markers for the identified cell types. Following this, we isolated the cancer epithelial cells and performed pseudotime trajectory analysis using Monocle. This analysis revealed three cell states originating from a single branch point. With increasing pseudotime, cells transitioned from state 1 to states 2 and 3 (**Figure 8D**). Notably, SHCBP1 expression rose as pseudotime increased and was more expressed in states 2 and 3 compared with state 1 (**Figure 8E**). We annotated cell states with canonical markers, such as CD44, PCNA, and MKI67 (**Figure 8F**). We found that state 1 is CD44-high, and states 2 and 3 were PCNA-high and MKI67-high. Therefore, state 1 was stem-like, whereas states 2 and 3 were proliferative.

**Figure 9:**
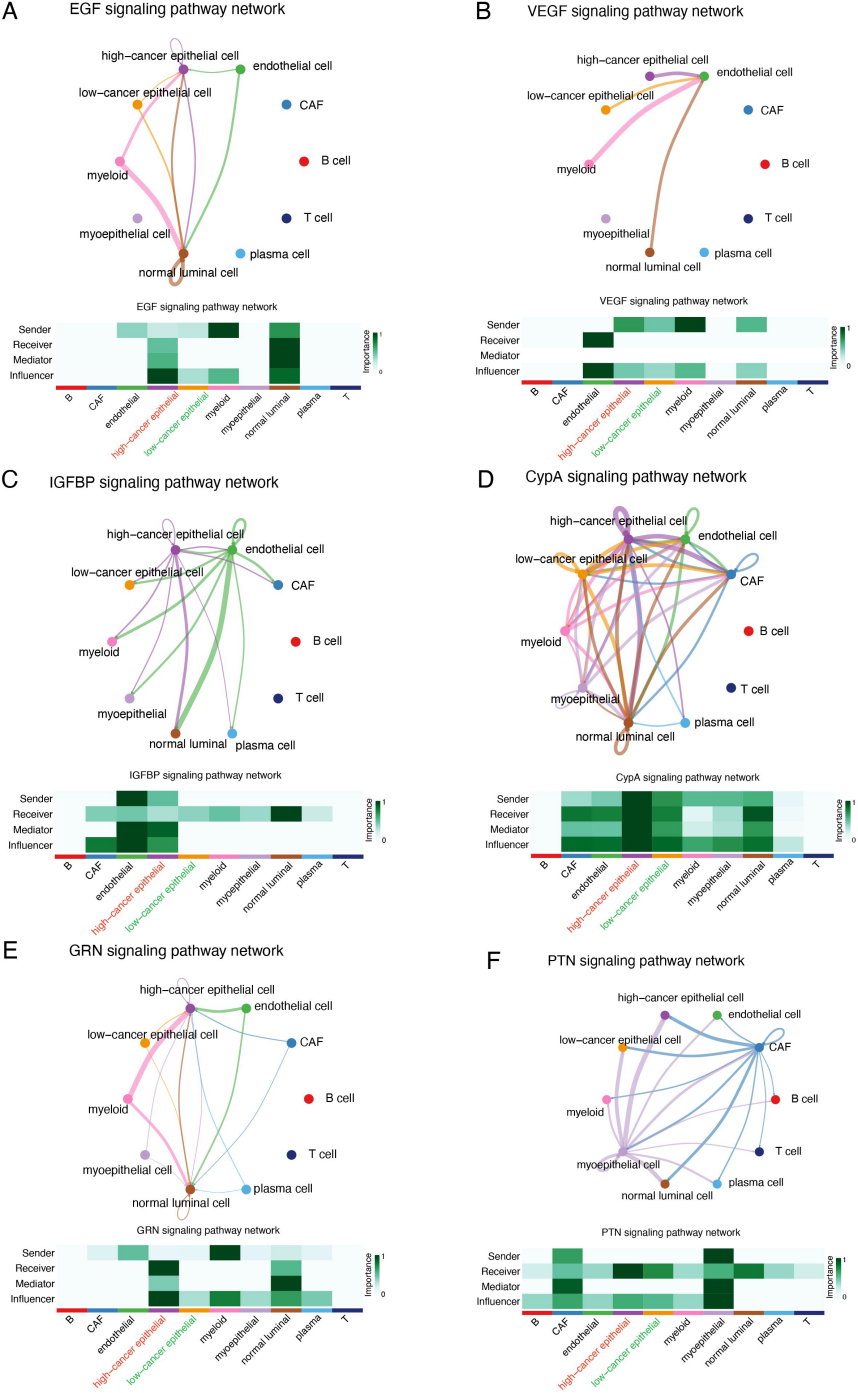
Cellular interaction analysis by CellChat. The cellular interaction network identified cell clusters in various signaling pathways, including **(A)** EGF, **(B)** VEGF, **(C)** IGFBP, **(D)** CypA, **(E)** GRN, and **(F)** PTN.

We divided cancer epithelial cells into high and low SHCBP1 clusters based on the median SHCBP1 expression. The high-SHCBP1 epithelial cluster exhibits elevated MKI67 (*p*<0.001), PCNA (*p*<0.001), and TOP2A (*p*<0.001), suggesting a higher proliferative epithelial state compared with the low-SHCBP1 epithelial cluster (**Supplementary Figure S1**). Subsequently, we calculated the cell–cell interactions among 10 cell types. The relationship between SHCBP1 expression and specific signaling pathways was further clarified. We found that cancer epithelial cells with elevated SHCBP1 expression exhibited a strong interaction with stromal cells through the EGF, VEGF, IGFBP, CypA, GRN, and PTN signaling pathways (**Figure 9**). These findings suggested that SHCBP1 may promote tumor progression and metastasis via the interaction of cancer epithelial cells with stromal cells.

## Discussion

In this research, we performed bioinformatics analysis and *in vitro* experiments to deeply explore the potential roles of SHCBP1 in TNBC. Our bioinformatics analysis revealed that SHCBP1 expression was significantly increased in TNBC, particularly in metastatic tissues. Elevated SHCBP1 levels correlated with a worse prognosis for TNBC patients. Functional enrichment analysis indicated that SHCBP1 is linked to cell cycle, microtubule binding, homologous recombination, and DNA damage. Therefore, we conducted *in vitro* experiments confirming that SHCBP1 promotes the proliferation and migration of TNBC cells. Moreover, we examined SHCBP1 expression patterns and its effects on cell communication within the TNBC at the single-cell level. In summary, our study is the first to systematically characterize the expression and function of SHCBP1 in TNBC, potentially paving the way for more detailed investigations into SHCBP1’s roles in TNBC.

SHCBP1 plays a crucial role in various cellular processes during cell division. Senga et al. reported that Aurora B phosphorylates SHCBP1 to promote the inactivation of Rac1 by MgcRacGAP and induce cytokinetic furrow ingression in HeLa cells (11). Moreover, SHCBP1 depletion promotes midbody structure disruption and inhibits abscission, a final stage of cytokinesis (12). Consistently, we found that SHCBP1 peaked in the M phase and co-localized with the spindle and centrosome during metaphase and anaphase in TNBC cells.

Accumulating evidence has demonstrated the abnormally high expression of SHCBP1 and its oncogenic role in multiple cancers (13), including gastric cancer (7), bladder cancer (6), lung cancer (8, 14, 15), pancreatic cancer (16), prostate cancer (17), and gliomas (18, 19). SHCBP1 is known to regulate tumor development by facilitating cell-cycle progression, augmenting cell survival, and mediating signal transduction (5). In lung cancer, EGF-induced nuclear translocation of SHCBP1 enhances β-catenin transactivation, increasing cellular stemness in non-small cell lung cancer (NSCLC) (14). Similarly, FGF13 interacts with SHCBP1 to activate AKT-GSK3α/β signaling and facilitate the cell cycle progression of A549 cells (20). In bladder cancer, nuclear translocation of SHCBP1 induced by EGF inhibits RACGAP1-mediated RAC1 inactivation to promote cancer cell proliferation and invasiveness (6). In gastric cancer, SHCBP1 interacts with PLK1 to enhance MISP phosphorylation, regulating trastuzumab sensitivity (7). In head and neck squamous cell carcinoma (HNSCC), SHCBP1 cooperates with KIF23 to regulate cell-cycle progression through several oncogenic signaling pathways (21). The mechanisms by which SHCBP1 regulates lung cancer progression were also elucidated by some researchers (22). Zhou et al. reported that SHCBP1 knockdown caused G2/M checkpoint impairment mediated by downregulated WEE1 kinase and NEK7 expression and upregulated centromere/kinetochore protein ZW10 expression. Despite these findings, the role of SHCBP1 in breast cancer, especially in TNBC, remains poorly understood. In our current study, we revealed a significant upregulation of SHCBP1 in TNBC and its association with a worse prognosis. Furthermore, our functional assays suggested that SHCBP1 enhanced the proliferation and migration of TNBC cells.

To validate the oncogenic role of SHCBP1 in TNBC at the single-cell level, we performed scRNA-seq analysis. As expected, the expression of SHCBP1 was higher in cancer epithelial cells compared with normal luminal cells. The high-SHCBP1 epithelial cluster exhibits elevated MKI67, PCNA, and TOP2A, suggesting a higher proliferative epithelial state compared with the low-SHCBP1 epithelial cluster. Additionally, the signaling pathways involved in the cell communications between the cancer epithelial cells and stromal cells differed regarding different SHCBP1 expression levels. Of note, the EGF, VEGF, IGFBP, CypA, GRN, and PTN signaling pathways were previously proven to be oncogenic signaling pathways (23–28). These pathways were more closely related to cancer epithelial cells with high SHCBP1 expression, further supporting the tumorigenic role of SHCBP1.

There are some limitations in this study. First, we only conducted preliminary *in vitro* functional experiments; the molecular mechanisms through which SHCBP1 exerts its oncogenic effects remain to be fully elucidated. Second, the translational relevance of our findings requires further validation through *in vivo* animal models and clinical specimen analysis. Despite these limitations, our study establishes a foundational framework for elucidating the molecular mechanisms of SHCBP1 and developing novel therapeutic strategies targeting SHCBP1 for TNBC patients.

## Conclusions

In conclusion, our study focused on the oncogenic and prognostic functions of SHCBP1 in TNBC using bulk RNA-seq, single-cell RNA-seq, and experimental data. We determined the upregulated expression and prognostic value of SHCBP1. We also demonstrated that SHCBP1 enhances TNBC cell proliferation and migration. Our findings will provide critical insights into the therapeutic potential of SHCBP1 as a molecular target in TNBC.

## Materials and methods

### Data gathering

The RNA-seq data and clinical information of 113 normal and 132 TNBC tissues were obtained from The Cancer Genome Atlas (TCGA) dataset. The transcriptomic profiles and clinical information of patients with TNBC were obtained from the National Omics Data Encyclopedia (NODE) (https://www.biosino.org/node/) (FUSCC, Project ID: OEP000155). The single-cell RNA sequencing data for TNBC samples were sourced from the Gene Expression Omnibus (GEO) database (https://www.ncbi.nlm.nih.gov/geo/query/acc.cgi?acc=GSE161529). Eight TNBC samples in GSE161529 were included in our study. Additionally, the Human Protein Atlas (HPA) database was used to examine SHCBP1 protein levels.

### Data preprocessing

The high-quality cells were acquired by following the procedures. (1) nFeature_RNA > 500; (2) percentage_mito ≤15%. A total of 58,804 cells were included for further analysis. The harmony package (V1.2.3) was employed to eliminate the batch effect of different samples. Clustering was performed using the Seurat (V5.1.0) functions FindNeighbors and FindClusters (resolution = 0.3). Clusters were then visualized with UMAP (29). The cell markers used for cell identification were obtained from previous research (30, 31).

### Single‐cell analysis

The CancerSEA online database (32) (http://biocc.hrbmu.edu.cn/CancerSEA/home.jsp) was utilized to investigate the role of SHCBP1 in breast cancer at a single-cell resolution. The CellChat package (V2.1.2) was employed to infer the communication network between cancer epithelial cells and stromal cells in TNBC. We used the netVisual circle plot in CellChat to visualize the differences in immune communication networks. Monocle 2 (V2.32.0) was applied to infer cell trajectories for cancer epithelial cells using the default parameters. The DDRTree method was performed for dimensionality reduction, with the max component set to 2.

### TNMplot database analysis

The TNMplot online tool (https://www.tnmplot.com/) was used to compare the expression of SHCBP1 in normal breast tissues, breast cancer tissues, and metastatic breast cancer tissues. The expression of SHCBP1 in pan-cancer tissues and their corresponding normal tissues was also analyzed by this tool.

### Differential expression and functional enrichment analyses

Expression profiles (HTSeq-Count) of TNBC were downloaded from the TCGA database. Differentially expressed genes (DEGs) between the high and low SHCBP1 expression groups were analyzed by the limma (V3.60.6) package. Differences with a |log2 fold change| > 0.6 and an adjusted *p*-value < 0.05 were considered significant. GO and KEGG analyses were performed using the DAVID database (33). GSEA was performed using the GSEA function in the “clusterProfiler” (V4.12.6) R package (34) and the HALLMARK and KEGG gene sets in the MSigDB (35) database. Gene set permutations were performed 1,000 times.

### Genetic alteration analysis

The cBioPortal database (36) (https://www.cbioportal.org/) was used to assess the genetic alteration frequency and mutation site of SHCBP1 in breast cancer. The Catalog of Somatic Mutations in Cancer (COSMIC) (37) (https://cancer.sanger.ac.uk/cosmic) database was utilized to identify the mutation types of SHCBP1 in TNBC. The single-nucleotide variant (SNV) data for TNBC from TCGA were downloaded using the R package “TCGAbiolinks” (V2.32.0). The R package “maftools” (V2.20.0) (38) was employed to visualize the mutation landscape of different groups.

### Cell culture and transfection

MCF10A and breast cancer cell lines (MCF7, T47D, BCAP37, BT549, MDA-MB-231, and BT-20) were obtained from Renji Hospital, Shanghai Jiaotong University, School of Medicine. MCF7, T47D, and BT-20 were cultured in DMEM, 10% fetal bovine serum (FBS), and 1% penicillin/streptomycin. BCAP37, BT549, and MDA-MB-231 were cultured in RPMI-1640, 10% FBS, and 1% penicillin/streptomycin. All cells were maintained at 37°C in a 95% air and 5% CO_2_ atmosphere.

The specific small interfering RNA targeting SHCBP1 (siSHCBP1) and negative control siRNA (siNC) were synthesized by the Shanghai GenePharma biotech company. The siRNA sequence was as follows: siNC forward, 5′-UUCUCCGAACGUGUCACGUTT-3′ and siNC reverse, 5′-ACGUGACAC GUUCGGAGAATT-3′; siSHCBP1–1 forward, 5′-GAGGAGAGUUACAGGAAAUTT-3′ and siSHCBP1–1 reverse, 5′-AUUUCCUGUAACUCUCCUCTT-3′; siSHCBP1–2 forward, 5′-GGUGCUGGUAUAGAAAUCUTT-3′ and siSHCBP1–2 reverse, 5′-AGAUUUCUAUACCAGCACCTT-3′. We transfected the siRNA into TNBC cells to knock down gene expression using jetPRIME^®^ *in vitro* DNA and siRNA transfection reagent following the manufacturer’s instructions. After 48 h, the knockdown efficiency was tested by qPCR or western blot.

Negative control and recombinant plasmid vectors overexpressing SHCBP1 (Asia-Vector Biotechnology) were constructed to transfect cells using jetPRIME^®^ *in vitro* DNA and siRNA transfection reagent following the manufacturer’s instructions. After 48 h, the overexpression efficiency was tested by qPCR.

### Agents and antibodies

Antibodies targeting SHCBP1 (Cat. no. 12672-1-AP) and GAPDH (Cat. no. 60004-1-Ig) were sourced from Proteintech. Antibody targeting β-Actin (Cat. no. ab49900) was purchased from ABCAM. Goat Anti-Rabbit IgG H&L (HRP) (Cat. no. A0208) and Goat Anti-Mouse IgG H&L (HRP) (Cat. no. A0216) were purchased from Beyotime. Nocodazole (HY-13520) was acquired from Selleckchem and MedChemExpress, respectively.

### Reverse transcription-quantitative polymerase chain reaction

Total RNA was extracted from TNBC cells by using SimplyP Total RNA Extraction Kit (BioFlux, USA) and was quantified by NanoDrop™ 2000/2000c Spectrophotometers (Thermo Scientific). RNA was reverse transcribed into cDNA using PrimeScript™ RT Master Mix (Cat. no. RR036A, TaKaRa). qPCR was performed by using 2× Universal Blue SYBR Green qPCR Master Mix (Cat. no. G3326-01, Servicebio) in the LightCycler 480 II instrument (Roche). Primer sequences are shown as follows: SHCBP1 forward, 5′-TGTCATTCAGGAGCAGGTTGTTCA-3′ and SHCBP1 reverse, 5′-TCACAGCACCACATCACACTTATT-3′; 18S forward, 5′-TGCGAGTACTCAACACCAACA-3′ and 18S reverse, 5′-GCATATCTTCGGCCCACA-3′. SHCBP1 relative expression was calculated against 18S expression by the 2^−ΔΔCt^ method.

### Western blot

Total protein extraction was performed with RIPA lysis buffer, followed by centrifugation at 12,000 rpm for 10 min at 4°C. The protein concentration was quantified by the BCA assay, and then the samples were boiled for 10 min in 5× SDS-PAGE loading buffer. 20 μg of total protein lysate was loaded on a 10% SDS-PAGE gel (Bio-Rad) and transferred onto PVDF membranes. Membranes were blocked in 5% skim milk for 1 h at room temperature. Subsequently, the membranes were incubated with primary antibodies against SHCBP1 (Cat no. 12672-1-AP, dilution 1/1,000, Proteintech), GAPDH (Cat no. 60004-1-Ig, dilution 1/50,000, Proteintech), and β-actin (Cat no. ab49900, dilution 1/50,000, ABCAM) overnight at 4°C. The membranes were washed in TBST and incubated with secondary antibodies (Goat Anti-Rabbit IgG H&L (HRP), Cat. no. A0208, dilution 1/1,000, Beyotime; Goat Anti-Mouse IgG H&L (HRP), Cat. no. A0216, dilution 1/1,000, Beyotime) for 1 h at room temperature.

### Cell cycle analysis

Trypsinized cells were fixed overnight in 75% ethanol at −20°C and washed with PBS. DNA was subsequently stained with propidium iodide (PI) solution for 30 min. The cells were finally examined by flow cytometry. The acquired data were analyzed with ModFit LT6.0 software.

### Immunofluorescence

Cells were seeded on the coverslip in 24-well plates and fixed with 4% formaldehyde for 10 min at room temperature. Next, cells were cleaned two times using PBS and permeabilized with 0.5% Triton X-100 for 10 min. Blocking was performed with 5% BSA for 1 h at room temperature, followed by overnight incubation at 4°C with the following primary antibodies (San Ying Biotechnology, China): SHCBP1, 1:400; α-tubulin, 1:300. The next day, cells were incubated with fluorescence conjugated secondary antibody (Proteintech, 1:200) for 1 h and stained with 2 µg/mL DAPI (C1105, Beyotime) for 10 min to label nuclei. The immunofluorescence images were acquired using a confocal microscope (Leica).

### Cell counting kit-8 assay

Cells were seeded in 96-well plates at a density of 5 × 10^3^ cells per well in 100 μL of culture medium. Subsequently, 10 µL CCK-8 reagent (Share-bio, Shanghai) was added to each well and incubated for 1 h. Absorbance was measured at 450 nm using a microplate reader.

### Colony formation assay

After transfection, cells were seeded in 6-well plates (2 × 10^3^ cells per well) and cultured for 2 weeks at 37°C, with the medium refreshed every 3 days. Colonies were stained with 1% crystal violet, photographed, and counted.

### Transwell assays

50,000 cells suspended in 200 μL of serum-free medium were added to the upper chamber. The lower chamber was filled with 600 μL culture medium containing 10% FBS to induce migration. After 48 h, non-migrated cells were removed using a cotton swab. Migrated cells on the lower membrane surface were fixed with 4% paraformaldehyde, stained with 1% crystal violet, and counted under a microscope to assess migration.

### Wound healing assays

After the transfected cells reached 100% density in 6-well plates, a pipette tip was used to scrape the cell layer. Then, the medium was replaced with serum-free medium. To determine the migration rate, we applied the following formula: wound closure (%) = (initial scratch distance - final cell-free image distance)/initial scratch distance.

### EdU assays

Cell proliferation was evaluated using the BeyoClick™ EdU Cell Proliferation Kit with Alexa Fluor 488 (C0071S, Beyotime).

### Survival analysis

The Kaplan–Meier method and Kaplan–Meier Plotter online tool (https://kmplot.com/analysis/) were employed to analyze the survival probability between high- and low-SHCBP1 groups in TNBC. The Kaplan–Meier analysis was performed to plot survival curves using the “survival” (V3.8-3) and “survminer” (V0.5.0) packages. All statistical tests were two-sided, with a significance level set at *p* < 0.05.

### Statistical analysis

Statistical analysis was conducted using R software (V4.4.1) and GraphPad Prism (V10.0). Differences between the two groups were assessed using Student’s t-test and the Wilcoxon test, with a *p*-value < 0.05 considered statistically significant.

## Data Availability

Publicly available datasets were analyzed in this study. This data can be found here: https://www.ncbi.nlm.nih.gov/geo/query/acc.cgi?acc=GSE161529).

## Supplementary material

Supplementary Figure 1 The violin plots compare the expression of proliferation markers (MKI67, PCNA, and TOP2A) in low-SHCBP1 and high-SHCBP1 cancer epithelial clusters. Data are presented as mean ± SD, ****p* < 0.001.
