# Supplementary figures and images for: SHCBP1 drives tumor progression in triple-negative breast cancer
Source: Front Oncol. 2025 Jul 29;15:1587236. doi: 10.3389/fonc.2025.1587236 (PMC12340242; doi:10.3389/fonc.2025.1587236)

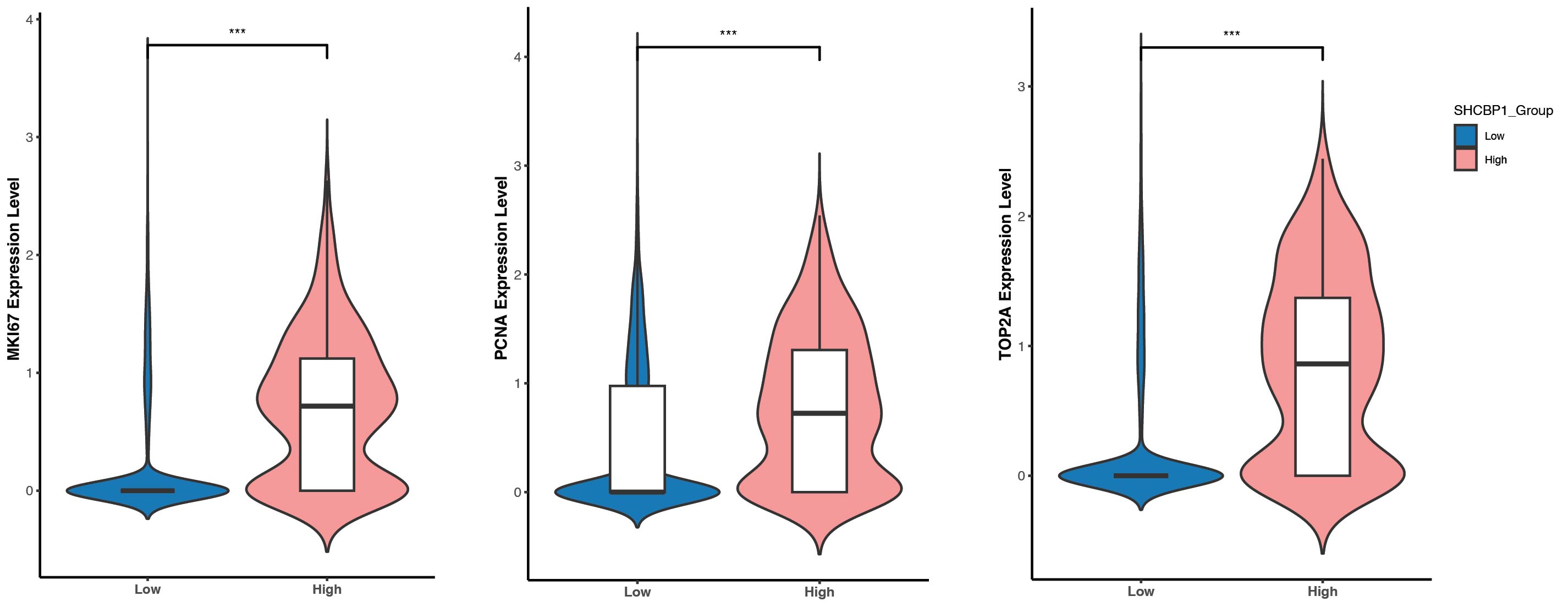

Supplement: Supplementary Figure 1 — The violin plots compare the expression of proliferation markers (MKI67, PCNA, and TOP2A) in low-SHCBP1 and high-SHCBP1 cancer epithelial clusters. Data are presented as mean ± SD, ***p < 0.001. [file Image1.jpg]
